# PRANA: an R package for differential co-expression network analysis with the presence of additional covariates

**DOI:** 10.1186/s12864-023-09787-3

**Published:** 2023-11-16

**Authors:** Seungjun Ahn, Somnath Datta

**Affiliations:** 1 Department of Population Health Science and Policy, Icahn School of Medicine at Mount Sinai, New York, USA; 2 Tisch Cancer Institute, Icahn School of Medicine at Mount Sinai, New York, USA; 3 Institute for Healthcare Delivery Science, Icahn School of Medicine at Mount Sinai, New York, USA; 4 Department of Biostatistics, University of Florida, Gainesville, USA

**Keywords:** Differential network analysis, Pseudo-value regression, RNA-Seq data, Covariate adjustment

## Abstract

**Background:**

Advances in sequencing technology and cost reduction have enabled an emergence of various statistical methods used in RNA-sequencing data, including the differential co-expression network analysis (or differential network analysis). A key benefit of this method is that it takes into consideration the interactions between or among genes and do not require an established knowledge in biological pathways. As of now, none of existing softwares can incorporate covariates that should be adjusted if they are confounding factors while performing the differential network analysis.

**Results:**

We develop an R package PRANA which a user can easily include multiple covariates. The main R function in this package leverages a novel pseudo-value regression approach for a differential network analysis in RNA-sequencing data. This software is also enclosed with complementary R functions for extracting adjusted *p*-values and coefficient estimates of all or specific variable for each gene, as well as for identifying the names of genes that are differentially connected (DC, hereafter) between subjects under biologically different conditions from the output.

**Conclusion:**

Herewith, we demonstrate the application of this package in a real data on chronic obstructive pulmonary disease. PRANA is available through the CRAN repositories under the GPL-3 license: https://cran.r-project.org/web/packages/PRANA/index.html.

## Background

The RNA-sequencing (RNA-Seq) leverages the rapid breakthroughs of the next-generation sequencing platform for profiling high-quality gene expression. Over the span of years, the RNA-Seq has emerged as an alternative to other gold standard techniques in transcriptomes [1, 2]. In contrast to microarrays, RNA-Seq achieves a higher resolution and lower technical variability [2–4] which leads to a higher reproducibility [5]. Another advantage of RNA-Seq relative to previously developed transcriptomic sequencing methods is that it has the ability to track transcriptomic dynamics (or gene expression changes) of tissues during physiological changes [5, 6], which thus allows a comparison of biological samples from patients with or without a specific disease or condition.

In response to these advantages, a vast number of statistical methods have become available to elucidate the genes or biological pathways associated with biological conditions or health outcomes, such as differential expression (DE) analysis [7, 8] and pathway enrichment (PE) analysis [9–11] of read counts (or gene expression) of an RNA-Seq data. However, it can be argued that results of DE analysis may provide limited information with the increased evidence that genes work in conjunction each other [12, 13]. The PE analysis appears to be a useful complement to the analysis of DE. The fundamental hypothesis in a PE analysis is that genes are regulated under common biological processes and clustered as a ‘pathway’ [13, 14], which borrows *a priori* pathway knowledge from the public repositories, namely, Gene Ontology [15], Kyoto Encyclopedia of Genes and Genomes [16], or Reactome [17]. To put it another way, PE analysis is primarily restricted to its use in a reference collection of well-studied biological processes only. Thereby, the idea of ‘network’ is introduced to pursue the veiled information that are obscured in those well-defined pathways [18].

The differential network (DN) analysis provides novel insights for identifying changes in gene-gene interactions under different biological conditions [19]. In theory, such changes are assessed through a comparison in characteristics of a network structure (*i.e.*, network topology) between two or more networks that are perturbed by a specific biological condition such as the development of cancer.

Despite the growing popularity, none of existing methods [20–22] fully addresses how to adjust for additional covariates (*e.g.* patient-age, patient-reported family histories, and other comorbidities) that may be associated with network topology.

Recently, we have adopted a pseudo-value regression [23] that allows covariate adjustment for the DN analysis while maintaining a high precision and recall values via a Monte Carlo simulation comparing with other methods available in R packages such as DINGO [20] and dnapath [22]. In addition, the computation time of this approach was shown competitive. To date, this is the first attempt of statistical method for the DN analysis with the inclusion of additional covariates.

In this article, we describe the software built as an R package, namely PRANA (**P**seudo-value **R**egression **A**pproach in **N**etwork **A**nalysis). PRANA is tailored to incorporate additional covariates information that may be associated with measures of connectivity of a gene (*i.e.* centrality) and a binary group indicator. This differs from previous statistical framework (or softwares) in DN analysis such as dnapath and DINGO.

## Implementation

### Algorithm

The algorithm below summarizes how the pseudo-value regression approach is embedded in a function named with PRANA. Briefly, the association measures are marginal quantities, such as degree centralities of each gene. Through the use of jackknife pseudo-values [24], we find the contribution of each individual data point to these quantities. Therefore, we could regress them on additional covariates as shown in studies with multi-state survival data [25, 26]. More details on methodological aspects are fully described elsewhere [23].

**Algorithm 1:** PRANA: Pseudo-value Regression Approach in Network Analysis

Input:	$n_z$ samples (in rows) × *p* expression levels of genes (in columns) RNA-Seq expression data and $n_z$ × *q* phenotype data for each group *z* = 1, 2.
Output:	A vector of adjusted p-values (and coefficient estimates and p-values) of the group variable for each gene *k* with a covariate adjustment.
1:	Estimate *p* × *p* association matrix (a matrix form of a network) via ARACNE [27] from the $n_z$ × *p* expression data for each group *z* = 1, 2.
2:	Obtain the group-specific degree centrality by taking the marginal sums of association matrix of each taxa *k* ∈ {1, · · · , *p*}.
3:	Repeat the first two steps above but using the association matrix that is re-estimated from the expression data without the *i* ∈ {1, · · · , $n_z$} individual of $n_z$ × *p* data.
4:	Calculate a group-specific jackknife pseudo-value for each gene *k* and individual *i* based on summary measures of degree centrality from Steps 2 and 3.
5:	For each gene *k*, a robust regression is fitted with a binary group variable and additional covariates to obtain the p-values of the group variable. In the regression, a binary group variable is the main predictor to declare a gene is DC between two groups under different conditions (or phenotypes).
6:	Lastly, a vector of gene-specific adjusted p-values [28] of the group variable is returned. See the Results section for more demonstration.

### Details of functions in PRANA

#### Main function

The main R function to perform the pseudo-value regression for the DN analysis with additional covariates is PRANA. The PRANA function imports two R scripts for the calculation of (1) total connectivity of an association matrix estimated from an observed expression data (as in thetahats function) and (2) adjusted *p*-values with the empirical Bayes screening procedure (as in EBS function) [28]. A list of three data.frame objects (coefficient estimates, *p*-values, and adjusted *p*-values of each predictor variable included in the regression for each gene) are returned upon the execution of PRANA function.

#### Supporting functions

For user convenience, we provide six additional R functions for extracting adjusted *p*-values (adjpval, adjpval_specific_var), coefficient estimates (coeff, coeff_specific_var), and genes that are significantly DC (sigDCGnames, sigDCGtab) from the output from PRANA function.

#### Dependencies

The PRANA package is fully implemented in R statistical programming language. The package depends on the base R packages (parallel, stats) and other R packages from the Comprehensive R Archive Network library (CRAN; dnapath, dplyr, robustbase) and Bioconductor (minet). Of important note, minet package should be directly installed from Bioconductor for a full utilization of PRANA package. This can be done by executing the code below in the R console.
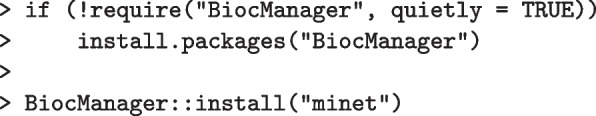


## Results

In this section, we illustrate how PRANA can be applied in practice using the sample dataset available from the package. This case study is the same as the one analyzed in our methodology paper [23].

### Sample dataset

The PRANA package includes a sample dataset named combinedCOPDdat_RGO with 406 samples. combinedCOPDdat_RGO consists of an RNA-Seq expression data for 28 genes that were spotlighted as associated with the chronic obstructive pulmonary disease (COPD) from a genome-wide association study [29]. It is a subset of the original study stored in the Gene Expression Omnibus (GEO) database with the accession number GSE158699 [30]. In this sample dataset, a phenotype data on six clinical and demographic variables is also available: current smoking status (main grouping variable), smoking pack years, age, gender, race, and FEV1 percent. The user can call the sample data into R by executing the following code:
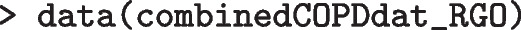


Alternatively, the user can also assign the data to an object by running the code below:
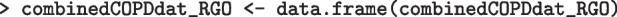


### Data processing

The PRANA function requires a user to provide each expression and phenotype data separately. 
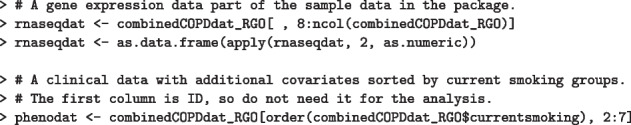


The main predictor variable in this example analysis is the current smoking status. As discussed in the Algorithm subsection, the estimation of association matrices (or networks) and the calculation of jackknife pseudo-values are carried out for each group separately. Hence, we add another step that locates the indices of subjects from each ‘current’ vs. ‘non-current smokers’ group. These indices are used to dichotomize expression dataset into ‘non-current smokers (Group A)’ and ‘current (Group B).’ 
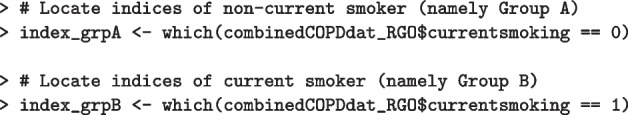


### Apply PRANA function for DN analysis with additional covariates

Once the data processing is complete, a user can perform a DN analysis with additional covariates. PRANA function takes an expression and phenotype data, separately, in which a user specifies each for RNASeqdat and clindat, respectively. To be more specific, the variables included in phenotype data are included in the regression. In addition, the group-specific indices for the main binary indicator variable are provided as groupA and groupB within the function. 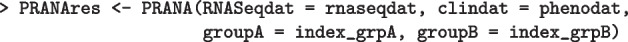


The output of the PRANA function is a list containing three data.frame objects for coefficient estimates, *p*-values, and adjusted *p*-values of all covariates included in the fitted model for each gene. Results are shown as following:
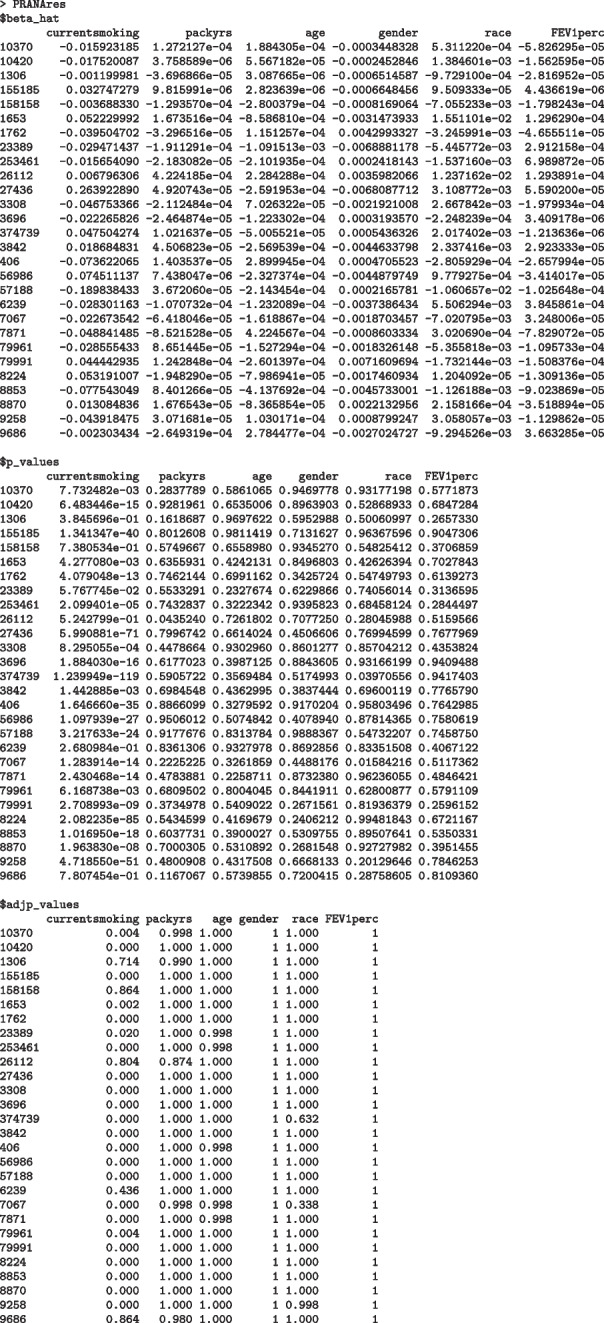


### Some supporting functions

The package offers some auxiliary features. A user can get a table of adjusted *p*-values and coefficient estimates for all variables with adjptab and coeff functions as following:
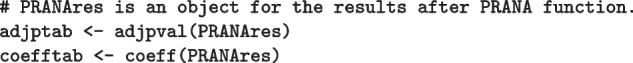


Suppose, for instance, we are interested in looking at the adjusted *p*-values for the current smoking status variable instead of a table with all variables. adjpval_specific_var function is available for that purpose:
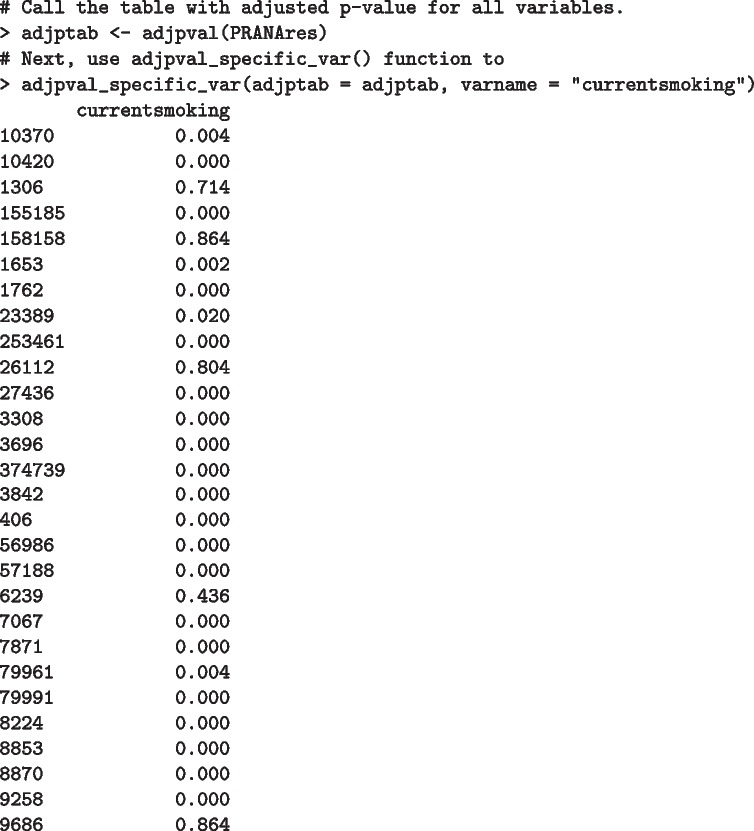


Similarly, coeff_specific_var function can be executed to return a coefficient estimate for a specific variable (current smoking status in the example below). A cautionary note is that the user must provide the name of a variable as in varname within each adjpval_specific_var or coeff_specific_var functions.
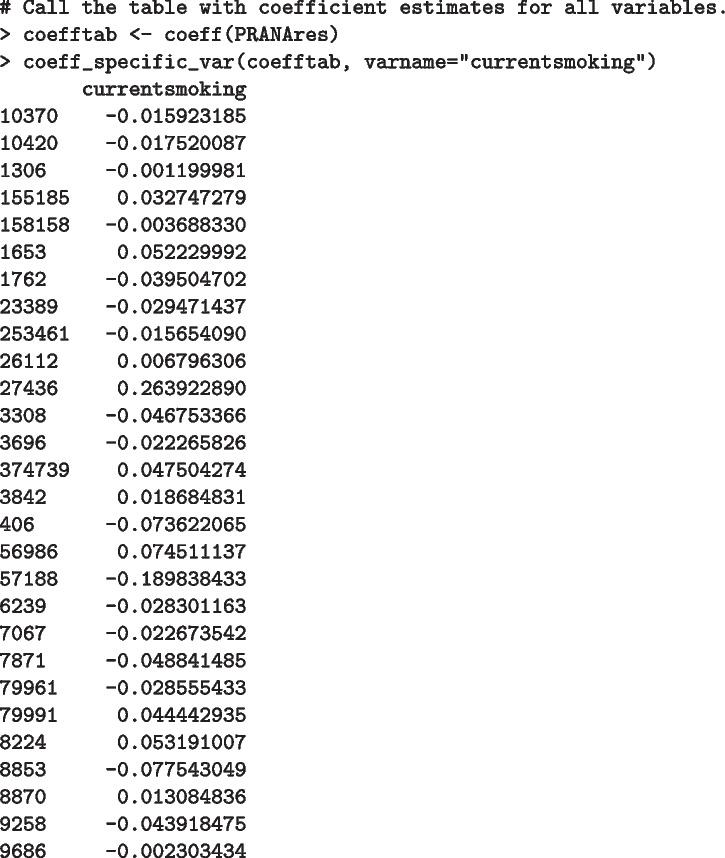


Additionally, sigDCGtab and sigDCGnames functions take a data.frame object as an input, defined by adjpval function earlier, to output the names of DC genes (*i.e.* NCBI Entrez gene IDs in the first column) for the main binary grouping variable utilized for the DN analysis, as well as corresponding adjusted *p*-values. sigDCGnames returns the names of DC genes only. A user may adjust the level of significance (alpha), which is set to 0.05 by default. Please see the following commands below:
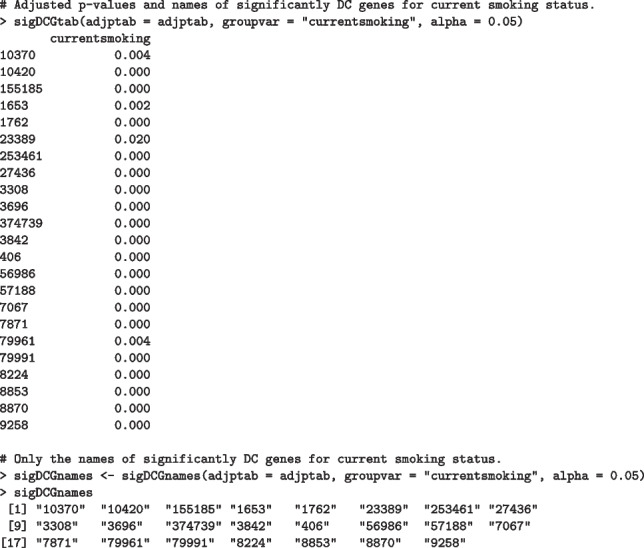


As a result, PRANA identified 23 genes that are significantly DC between current and non-current smokers while accounting for additional covariates such as smoking pack years, age, gender, race, and FEV1 percent.

As an additional step, a user can utilize rename_genes function from the dependency package (dnapath) to rename results with Entrez gene IDs into gene symbols. See below for the demonstration in R console. Results are summarized in Table 1.
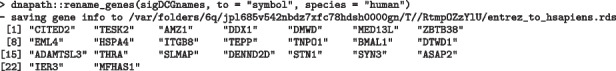

**Table 1:** Results of DC genes obtained from PRANA. The sample dataset contains the NCBI Entrez gene IDs, so does the resulted DC genes (first column). dnapath::rename_genes is utilized to rename Entrez gene IDs to gene symbol (second column)

Entrez ID	Gene symbol
10370	CITED2
10420	TESK2
155185	AMZ1
1653	DDX1
1762	DMWD
23389	MED13L
253461	ZBTB38
27436	EML4
3308	HSPA4
3696	ITGB8
374739	TEPP
3842	TNPO1
406	BMAL1
56986	DTWD1
57188	ADAMTSL3
7067	THRA
7871	SLMAP
79961	DENND2D
79991	STN1
8224	SYN3
8853	ASAP2
8870	IER3
9258	MFHAS1

## Discussion

The R package PRANA has been published in the CRAN (https://cran.r-project.org/web/packages/PRANA/index.html). This package has no operating system dependencies. A vignette is available on this package at https://cran.r-project.org/web/packages/PRANA/vignettes/UserManualPRANA.html or can be accessed by typing in an R console (browseVignettes(package="PRANA")). In this package, the sample dataset is provided with COPD-related genes, as well as clinical and demographic variables. The source code of the package can be found in GitHub: https://github.com/sjahnn/PRANA.

PRANA has some plans for future development. Firstly, although a user may attempt a classical regression-based variable selection such as stepwise selection, we have not yet validated this through a statistical simulation experiment. Secondly, the names of genes provided in the sample dataset are Entrez gene IDs. Further extension will include a function that convert from these gene IDS to gene symbols (*i.e.* 10370 to CITED2) and vice versa for user convenience.

In conclusion, PRANA is a user-friendly and novel regression-based method that accounts for additional covariates along with the main binary grouping variable for the DN analysis.

## Conclusions

The differential network analysis identifies changes in measures of associations between genes under different biological conditions. Although there has been increasing volume of work in this subject, overall covariate adjustment remains underexplored. In this paper, we present PRANA, the first R package that adjusts for additional covariates for the differential network analysis. As a brief note on the usage, PRANA takes RNA-sequencing and phenotype data (metadata) as inputs and in return tables containing DC gene names and their corresponding adjusted *p*-values are produced for a main binary grouping variable to be adjusted with the presence of additional covariates. This software is easy to install and user-friendly.

## Availability and requirements

**Project name**: PRANA

**Project home page**: https://cran.r-project.org/web/packages/PRANA/index.html

**Operating system(s)**: Platform independent

**Programming language**: R

**Other requirements**: Install dnapath, dplyr, robustbase, and minetR packages

**License**: GNU GPL-3

**Any restrictions to use by non-academics**: No restrictions

## Data Availability

PRANA is freely available at (https://cran.r-project.org/web/packages/PRANA/index.html). The COPDGene data is available in the GEO database with accession number GSE158699 (https://www.ncbi.nlm.nih.gov/geo/query/acc.cgi?acc=GSE158699). Please reach out to the maintainer (Seungjun Ahn, seungjun.ahn@mountsinai.org) if you have any further inquiries on the data or code.

## Abbreviations

COPD: Chronic obstructive pulmonary disease
DC: Differentially connected
DN: Differential network
GEO: Gene Expression Omnibus
